# Pharmacological Effects of Marine-Derived *Enterococcus faecium* EA9 against Acute Lung Injury and Inflammation in Cecal Ligated and Punctured Septic Rats

**DOI:** 10.1155/2021/5801700

**Published:** 2021-12-06

**Authors:** Hatem M. Abuohashish, Eman H. Zaghloul, Amany S. El Sharkawy, Eman M. Abbas, Mohammed M. Ahmed, Salim S. Al-Rejaie

**Affiliations:** ^1^Department of Biomedical Dental Sciences, College of Dentistry, Imam Abdulrahman Bin Faisal University, Dammam 31441, Saudi Arabia; ^2^National Institute of Oceanography and Fisheries (NIOF), Cairo 11516, Egypt; ^3^Department of Pharmacology and Toxicology, College of Pharmacy, King Saud University, Riyadh 11451, Saudi Arabia

## Abstract

Microorganisms obtained from the marine environment may represent a potential therapeutic value for multiple diseases. This study explored the possible protective role of marine-derived potential probiotic *Enterococcus faecium* EA9 (*E. faecium*) against pulmonary inflammation and oxidative stress using the cecal ligation and puncture (CLP) model of sepsis in Wistar rats. Animals were pretreated with *E. faecium* for 10 days before either sham or CLP surgeries. Animals were sacrificed 72 hours following the surgical intervention. The histological architecture of lung tissues was evaluated as indicated by the lung injury score. In addition, the extend of pulmonary edema was determined as wet/dry weight ratio. The inflammatory cytokines were estimated in lung tissues, including tumor necrosis factor-alpha (TNF-*α*), interleukin-6 (IL-6), and interleukin-1 beta (IL-1*β*) using the enzyme-linked-immunosorbent-assay (ELISA) technique. Moreover, markers for lipid peroxidation such as thiobarbituric acid reaction substances (TBARs), and endogenous antioxidants, including reduced glutathione (GSH) were determined in lung tissues. Finally, the enzymatic activities of antioxidant enzymes such as catalase (CAT), superoxide dismutase (SOD), glutathione peroxidase (GPx), and glutathione reductase (GR) were assayed in the lungs. Pretreatment with *E. faecium* markedly attenuated CLP-induced lung injury and pulmonary edema. Markers for inflammation, including TNF-*α*, IL-6, and IL-1*β* were augmented in the lung tissues of CLP animals, while *E. faecium* ameliorated their augmented levels. *E. faecium* pretreatment also restored the elevated TBARS levels and the prohibited CAT, SOD, and GPx enzymatic activities in CLP animals. GSH levels were corrected by *E. faecium* in CLP animals. The inflammatory and lipid peroxidation mediators were positively correlated, while antioxidant enzymatic activities were negatively correlated with CLP-induced lung injury and pulmonary edema. Collectively, marine-derived *Enterococcus faecium* EA9 might be considered as a prospective therapeutic tool for the management of pulmonary dysfunction associated with sepsis.

## 1. Introduction

Sepsis is a life-threatening condition in which the human body responds to infection that injures the internal organs leading to their dysfunction. This condition might progress to septic shock. The prevalence of sepsis is high despite the advances in medical services and intensive care unit settings. Studies showed that more than 1 million deaths might occur around the world due to sepsis particularly in the neonatal population [1, 2]. Sepsis also is associated with low-birth-weight infants and long-term adverse consequences. Lungs are one of the most affected organs in sepsis. Sepsis might result in acute lung injury and acute respiratory distress (ARDS), which is a common challenge that triggers the mortality rate especially in pediatric patients [3, 4]. Studies demonstrated that mortalities due to acute lung injury/ARDS are around 26% in the United States [5]. Experimental sepsis models include the cecal ligation and puncture (CLP) model. This model is extensively used in rodents to mimic the intensive production of inflammatory mediators such as reactive oxygen species (ROS) and cytokines, which altered respiratory function resulting in pulmonary edema and inflammatory exudate accumulation [6].

Probiotics are nonpathological bacteria that live in and colonize the human intestine. They are a useful tool for the host to counteract the detrimental effects of altered or pathological flora. Several randomized controlled trials introduced probiotics for the management of nosocomial infections, including alimentary tract infections as well as respiratory tract infections in adult and pediatric patients [7–9]. In addition, the use of probiotics offers an advantage over conventional antimicrobial interventions as it has lower antimicrobial resistance. In the last two decades, probiotics were introduced as a therapeutic tool in the treatment of sepsis. Clinical evidence demonstrated that probiotics decrease late-onset sepsis incidence in preterm infants [10]. In addition, supplementation of children suffering from severe sepsis with probiotics for 7 days significantly lowered the levels of inflammatory mediators in one randomized controlled trial [11]. The use of probiotics in experimental animals also attenuated sepsis-associated inflammation and oxidative injuries [12, 13]. Interestingly, the use of probiotics such as *Lactobacillus rhamnosus* GG and *Bifidobacterium longum* [14] and *Lactobacillus fermentum* [15] was able to reverse the pulmonary dysfunction and inflammation using CLP and lipopolysaccharide (LPS) models of sepsis, respectively.

Probiotic strains such as *Lactobacilli*, *Bifidobacteria*, and *Streptococcus thermophilus* are present in several nutritional products like fermented and unfermented milk, yogurts, some juices, and soy beverages [16]. Currently, there are several attempts to develop probiotics from the marine environment as it represents a potential source of novel microorganisms. These microorganisms may produce a vast diversity of secondary metabolites and bioactive molecules, which cannot be acquired from nonaquatic microorganisms [17]. Moreover, marine lactic acid bacteria have potential applications in different industrial and pharmaceutical perspectives along with their significant environmental role in the transformation of organic materials in marine sediments [18]. In this context, this study is aimed at exploring the possible protective role of marine-derived potential probiotic *Enterococcus faecium* EA9 (*E. faecium*) against lung dysfunction and inflammation in the CLP model of sepsis.

## 2. Materials and Methods

### 2.1. Animals and Ethical Approval

In this study, twenty-four male Wistar albino rats were employed. Experimental animals were kindly provided from the animal house at the Institute of Graduate Studies and Research, Alexandria University, where all surgical procedures, including euthanasia, were conducted. Rats were maintained under a controlled environment, including the acclimatization and experimental treatment periods. All investigational conditions and surgical procedures were conducted in agreement with the National Institute of Health (NIH) guide for the care and use of laboratory animals “8th edition.” Moreover, the experimental protocol of the present study was also ethically approved by the Institutional Animal Care and Use Committee (IACUC) at Alexandria University (AU14-210126-3-2).

### 2.2. Experimental Model and Design

Animals were randomly allocated into four groups as follows: (a) sham animals with no CLP or probiotic treatment (Sham), (b) sham animals with probiotic treatment (Sham+*E. faecium*), (c) CLP animals with no probiotic treatment (CLP), and (d) CLP animals with probiotic treatment (CLP+*E. faecium*). The surgical procedure of the CLP model of sepsis was conducted according to Zubrow et al. [19]. In brief, animals were generally anesthetized using a single intraperitoneal injection of ketamine (EIPICO, 10^th^ of Ramadan city, Egypt) and xylazine (Adwia, 10^th^ of Ramadan city, Egypt) mixture (60 and 5 mg/kg, respectively). Then, a median laparotomy incision of 15 mm long was made, and the cecum was visualized and exposed. The distal section of the isolated ileocecal valve/cecum was ligated using a 4-0 silk thread. The bowel continuity was maintained and not disrupted during the ligation process. After ligation, the ligated portion of the cecum was subjected to multiple (2 to 3) punctures using an 18-gauge syringe needle. Then, it was squeezed to extrude an insignificant volume of feces to assure the opening at the puncture site. At the end of the surgical procedure, the ligated cecum was returned to the abdominal cavity. Sham animals had the same surgical procedure except for the ligation and puncture steps. All animals were provided sterile saline (20 ml/kg body weight) subcutaneously. Postoperative pain was maintained using intraperitoneal injection of ketorolac (30 mg/kg) every 12 h (Amriya Pharmaceutical Industries, Amriya, Egypt). The marine isolate *Enterococcus faecium* EA9 (GenBank: MW218438.1) was isolated from the gut of shrimp samples collected from the Mediterranean Sea, Egypt. It was isolated on MRS Agar (De Man, Rogosa and Sharpe) medium (LabM, UK) and further evaluated for having potential probiotic properties (under processing data). The freeze-dried probiotic was resuspended in 1 M NaCl (1 × 10^7^ CFU/ml), and a 250 *μ*l was given immediately, by oral gavage for 10 days before the CLP surgeries, to each rat in sham+*E. faecium* and CLP+*E. faecium* groups. 72 hours after the CLP procedure, animals were euthanized using a high dose of inhaled general anesthesia (Aesica Queenborough Ltd., Kent, UK). Lung samples were collected for analysis. The lower lobe of the right lung was used in pulmonary edema estimation, while the remaining section of the right lung was preserved in 10% formalin solution for histological examination. The left lung samples were stored in -20°C for biochemical analysis.

### 2.3. Estimation of Pulmonary Edema

The extent of pulmonary edema was determined in the extracted lung tissues by measuring wet/dry. For each animal, the lower lobe of the right lung was isolated and weighed. This was considered the wet weight. Then, the lobes were dried at 60°C for 24 hours. The dried lobes were reweighed to calculate the wet/dry ratio.

### 2.4. Histological Examination

The right lung of each rat was fixed in a 10% formalin solution (Sigma-Aldrich, MO, USA). Samples were embedded in paraffin blocks and sectioned using a microtome. Afterward, the sectioned samples were stained with hematoxylin and eosin (Sigma-Aldrich, MO, USA). Slides were examined under a microscope (Leica Biosystems Melbourne Pty Ltd., Melbourne, Australia) and scored blindly as previously described [20]. Lung injury was given a score out of 100 based on the occurrence of inflammatory infiltration from neutrophils, structure of the hyaline membrane, airspaces, and septal thickening.

### 2.5. Detection of Pulmonary Oxidative Stress Biomarker

The left lung samples were homogenized in a physiological buffer (1 : 10, *w*/*v*). Thiobarbituric acid reactive substance (TBARS) level was determined, as a marker for lipid peroxidation, while reduced glutathione (GSH) level was measured, as a marker for endogenous antioxidant, using commercially diagnostic kits (Cayman Chemical Co., USA). The homogenate was then centrifuged to produce the postmitochondrial supernatants. Enzymatic activities of antioxidant enzymes such as catalase (CAT), superoxide dismutase (SOD), glutathione reeducates (GR), and glutathione peroxidase (GPx) were estimated by assay kits (R&D Systems Inc., USA).

### 2.6. Detection of Pulmonary Inflammatory Cytokines

In the left lung tissue homogenates, the levels of inflammatory cytokines, including tumor necrosis factor-alpha (TNF-*α*), interleukin-6 (IL-6), and interleukin-1 beta (IL-1*β*) were quantitatively determined using the enzyme-linked-immunosorbent-assay (ELISA) technique following instructions provided by the kits (R&D Systems Inc., USA).

### 2.7. Statistical Analysis

The numerical values of the results were presented in the form of the mean of each group ± standard deviation of the mean (SD). One-way analysis of variance (ANOVA) was used as a statistical analysis test to evaluate the difference between the four groups. One-way ANOVA was followed by the Student-Newman-Keuls post hoc test. Data were analyzed using GraphPad Prism 5 (GraphPad Software, Inc., La Jolla, CA, USA). The difference between the means was considered significant when *p* values were equal to or less than 0.05.

## 3. Results

**Figure 1:**
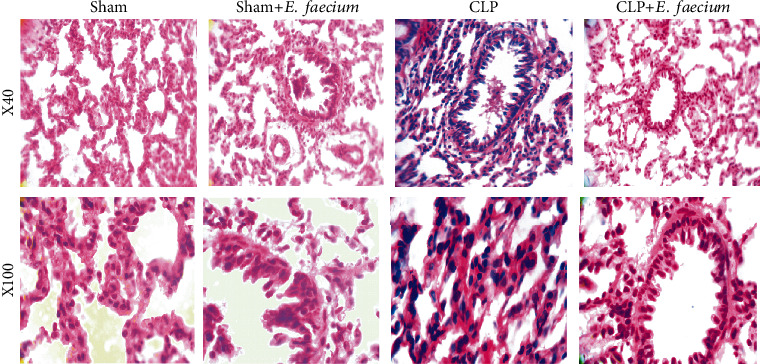
Histological evaluation of marine *E. faecium* effects on pulmonary inflammation and injury in Wistar rats with sepsis. Images are represented in ×40 and ×100 magnifications. The sham group showed regular lung architecture with no inflammatory exudates and normal hyaline membranes and septal thickening. Similarly, the sham+*E. faecium* group demonstrated ordinary histological features of lung tissues without inflammation or altered hyaline membranes and septal thickening. In the CLP group, there was a significant inflammatory infiltration of neutrophils and hyaline membranes with marked thickening of the septum. The CLP+*E. faecium* group revealed low extent of neutrophilic inflammatory infiltrate with mild septal thickening and hyaline deposition.

Results of the histological investigation in lung tissues showed that animals in the sham group have a normal appearance and histological features. There were no signs of inflammatory infiltration or thickening of the septum. Sham animals with *E. faecium* pretreatment also had normal lung histology without inflammation or thickening as compared to sham animals. However, animals in the CLP group had marked inflammation infiltration and altered hyaline membrane. Pretreatment of CLP animals with marine-derived *E. faecium* markedly restored the histological characteristics of lung tissues, lowered the neutrophilic inflammatory infiltration, and attenuated septal thickening and deposition of the hyaline membrane (Figure 1).

**Figure 2:**
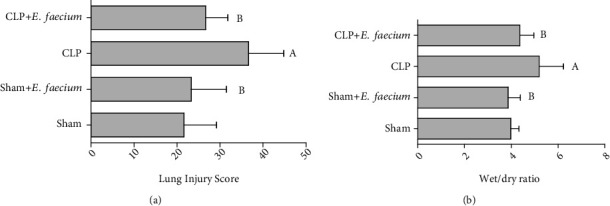
Effects of marine *E. faecium* pretreatment in Wistar rats with sepsis on (a) lung injury score determined by histological analysis and (b) pulmonary edema expressed as wet/dry ratio. Data are presented as mean ± SD (*n* = 6) and statistically analyzed by one-way ANOVA followed by the Student-Newman-Keuls post hoc test. Statistical significance was considered when *p* ≤ 0.05. Statistical significance was considered when “A” vs. sham group and “B” vs. CLP group.

Quantitative scoring of the histological slides revealed a significant (*p* ≤ 0.05) increase in the lung injury score in the CLP group as compared with the sham group. When comparing the CLP group with sham and CLP animals with *E. faecium* pretreatment, the CLP group showed a significant (*p* ≤ 0.05) higher lung injury score as compared to *E. faecium*-pretreated groups (Figure 2(a)). Pulmonary edema was assessed as a wet/dry ratio of lung tissues. The CLP group had a significant (*p* ≤ 0.05) high wet/dry ratio as compared to sham animals. Moreover, the wet/dry ratio was significantly lowered in sham animals and CLP animals who had pretreatment with *E. faecium* (*p* ≤ 0.05) as compared with the CLP group (Figure 2(b)).

**Figure 3:**
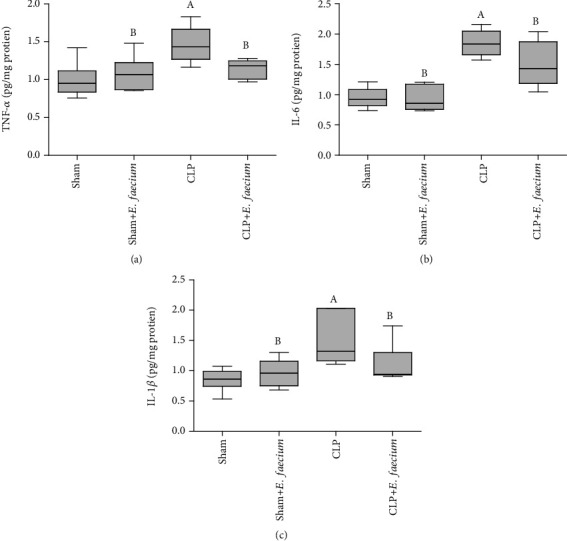
Effects of marine *E. faecium* pretreatment in Wistar rats with sepsis on lung levels of (a) tumor necrosis factor-alpha (TNF-*α*), (b) interleukin-6 (IL-6), and (c) interleukin-1 beta (IL-1*β*). Data are presented as mean ± SD (*n* = 6) and statistically analyzed by one-way ANOVA followed by the Student-Newman-Keuls post hoc test. Statistical significance was considered when *p* ≤ 0.05. Statistical significance was considered when “A” vs. sham group and “B” vs. CLP group.

Lung tissues of the sham group had significantly lower values of TNF-*α*, IL-6, and IL-1*β* (*p* ≤ 0.01, 0.001, and 0.01, respectively) as compared to the CLP group. These inflammatory mediators were low in the sham+*E. faecium* group (*p* ≤ 0.05, 0.001, and 0.05, respectively) when compared to the CLP group. Pretreatment of CLP animals with *E. faecium* significantly reduced the lung levels of TNF-*α*, IL-6, and IL-1*β* (*p* ≤ 0.05) as compared to CLP animals without *E. faecium* pretreatment (Figure 3).

**Figure 4:**
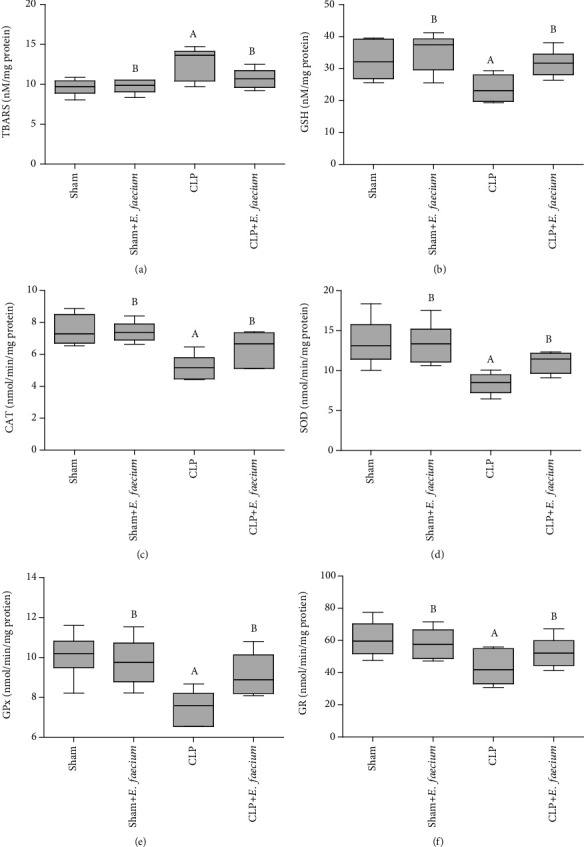
Effects of marine *E. faecium* pretreatment in Wistar rats with sepsis on lung levels of (a) thiobarbituric acid reaction substances (TBARs), and (b) reduced glutathione (GSH) as well as the enzymatic activities of (c) catalase (CAT), (d) superoxide dismutase (SOD), (e) glutathione peroxidase (GPx), and (f) glutathione reductase (GR). Data are presented as mean ± SD (*n* = 6), and statistically analyzed by one-way ANOVA followed by the Student-Newman-Keuls post hoc test. Statistical significance was considered when *p* ≤ 0.05. Statistical significance was considered when “A” vs. sham group and “B” vs. CLP group.

TBARS was assessed in the lung tissues as a marker for lipid peroxidation. TBARS levels in the lung tissues of the CLP group were significantly (*p* ≤ 0.01) higher than those in the sham group. Though, the lung levels of TBARS were significantly low in the sham and CLP groups with *E. faecium* pretreatment (*p* ≤ 0.01 and *p* ≤ 0.05, respectively) as compared to the CLP group. The endogenous antioxidant, GSH, was significantly (*p* ≤ 0.05) lower in the CLP group as compared with the sham group. Lung levels of GSH were higher in sham+*E. faecium* and CLP+*E. faecium* groups (*p* ≤ 0.01 and *p* ≤ 0.05, respectively) when compared to the CLP group (Figure 4). The enzymatic activities of antioxidant enzymes, including CAT, SOD, GPx, and GR, were significantly (*p* ≤ 0.001, *p* ≤ 0.01, *p* ≤ 0.01, and *p* ≤ 0.05, respectively) higher in the sham group as compared to the CLP group. Likewise, CAT, SOD, GPx, and GR activities were significantly (*p* ≤ 0.001, *p* ≤ 0.01, *p* ≤ 0.01, and *p* ≤ 0.05, respectively) higher in the sham+*E. faecium* group as compared to CLP group. Pretreatment of animals with CLP surgeries with *E. faecium* significantly (*p* ≤ 0.05) increased the enzymatic activities of CAT, SOD, and GPx (Figure 4).

Correlation analysis revealed a significant positive correlation between lung injury score and inflammatory cytokines, including IL-6 and IL-1*β* (*p* = 0.0047 and 0.0324, respectively). Lung injury score was negatively correlated with TBARS levels (*p* = 0.0135) and the enzymatic activities of CAT and SOD (*p* = 0.0004 and 0.0339, respectively) (Table 1). In addition, there was a marked positive correlation between the pulmonary edema and all inflammatory cytokines, including TNF-*α*, IL-6, and IL-1*β* (*p* = 0.0236, 0.0241, and 0.0162, respectively). The lung level of TBARS and the enzymatic activities of SOD, GPx, and GR were also correlated with pulmonary edema significantly (*p* = 0.0008, 0.0103, 0.0286, and 0.0270, respectively) (Table 2).

## 4. Discussion

Pulmonary dysfunction and acute respiratory distress are vital inflammatory complications in patients with sepsis, which result in an increased risk of mortality. Multiple pathogenic microorganisms have been involved in this critical condition. Accordingly, several therapeutic approaches have been introduced to regulate this pathological illness. One major objective is to treat pulmonary-associated inflammation and injury without resistance. In this context, probiotics have been evaluated clinically and experimentally. The prospective therapeutic effects of marine-derived *Enterococcus faecium* EA9 were evaluated in the present study against sepsis-induced lung inflammation and dysfunction. This study revealed that the probiotics obtained from the marine environment could restore pulmonary injury and edema after CLP surgery. In addition, the elevated markers of inflammation and oxidative stress were markedly corrected to their normal levels in the lungs of CLP animals following the pretreatment with *Enterococcus faecium* EA9.

The CLP model of sepsis has been widely used in rodents. Experimental evidence suggests the successes of this model. Pathogens might migrate from the intervention site and cause multiple internal organ infections, injuries, and dysregulations. This model showed marked alteration in pulmonary functions and induced marked inflammatory pathways. In Chen et al.'s [21] study, CLP remarkably injured young rats' lungs causing edema and apoptosis. Another experimental study conducted on Wistar rats using the same animal model showed that dexmedetomidine, a well-known anti-inflammatory, restored lung inflammation, edema, and apoptosis [22]. In the present study, CLP induced significant pulmonary injury and altered the normal histological features. The hyperinflammatory status in the lungs of CLP-operated animals that was characterized by a significant increase in inflammatory cytokines was also reported in other studies [23]. Moreover, the CLP model caused a significant pulmonary edema as indicated by the increased wet/dry ratio. Previous studies also documented comparable pulmonary edema using the same animal model [19, 24].

Reactive oxygen metabolites are considered crucial caustic mediators in the pathogenesis of lung injuries during sepsis [25]. Accordingly, the sepsis model in experimental animals may trigger pulmonary oxidative stress by limiting the endogenous antioxidant (GSH) levels and restricting the activities of antioxidant enzymes. These effects were reported in the present study along with marked lipid peroxidation. The elevated lung levels of TBARS in CLP animals indicate exaggerated cellular membrane damage and apoptosis due to the accumulation of ROS, which affects cellular fluidity and permeability resulting in dysfunction. SOD and CAT are essential antioxidant enzymes that scavenge ROS such as hydrogen peroxide and convert them into oxygen and water. Other antioxidant protective enzymes such as GPx and GR decrease the levels of lipid hydroperoxide and hydrogen peroxide. The CLP model restricts the antioxidant enzyme activities and triggers inflammatory cytokines in vital organs such as the kidney and heart [26]. Notably, studies have suggested that oxidative stress may play a vital role in the pathogenesis of sepsis-induced lung injury and edema. In Emel and Hilal's [27] study, CLP triggered pulmonary oxidative stress and lipid peroxidation along with a reduction in the activities of antioxidant enzymes, including the myeloperoxidase (MPO), SOD, and CAT. Furthermore, another study showed the association between acute lung injury in septic rats and cellular oxidative damages [28].

The health benefits of probiotics are documented, and accordingly, several biomedical applications were introduced [29]. For instance, probiotics might enhance the health conditions and meat production capabilities of broilers and goats [30, 31]. Probiotics also improve the sustainability and performance of marine animals [32]. The probiotic use to manage multiple organs' inflammatory response and cellular damage in the CLP model of sepsis has been introduced in several experimental studies. In one study, mice with sepsis-induced encephalopathy were treated with the probiotic *Clostridium butyricum*. This treatment alleviated the cognitive impairments and exerted neuroprotective effects [33]. *Lactobacillus rhamnosus* GG, as a probiotic, was able to reduce CLP-associated liver damages, hypoxic hepatitis, and inflammation in septic rats [34], while, in CLP septic C57BL6 mice, *Lactobacillus rhamnosus* GG enhanced intestinal permeability and ileum mucosal damage [35, 36]. Guo et al. reported that pretreatment with a mixture of *Bacillus subtilis* and *Enterococcus faecium* can reveal significant intestinal protective effects in septic mice *via* counteracting intestinal inflammation using the CLP model [37]. Interestingly and similar to our study, treatment of FVB/N mice with either *Lactobacillus rhamnosus* GG or *Bifidobacterium longum* markedly lowered lung inflammatory neutrophilic infiltrates following sepsis induction by the CLP model in Khailova et al.'s study [14]. Our findings are in agreement with the trend that probiotic treatment may represent a safe and less resistant approach in sepsis. The aquatic probiotic used in the present study reduced pulmonary hyperinflammation and restored lung injury, histological features, and edema.

Marine microorganisms have a wide taxonomic diversity. They live in a unique habitat subjected to extreme environmental conditions, which necessitate the development of different adaptation mechanisms to withstand these unusual surroundings. They develop a variety of novel secondary metabolites and bioactive compounds with different prospective and structures that cannot be obtained from terrestrial microorganisms [17]. Several attempts were made to produce probiotics from the marine environment. Different marine probiotic bacteria, which belong to phylum Proteobacteria such as *Pseudomonas*, *Shewanella*, and *Phaeobacter*, had an excellent impact on the growth and immune response of fish cultures [38]. One study showed that a marine isolate *Psychrobacter maritimus* S could enhance the growth and performance of Nile tilapia fingerlings fish [39]. Another study demonstrated that the marine probiotic, *Bacillus pumilus* H2, could provide marked preventative effects against fish vibriosis [40]. Aquatic probiotic strain, *Bacillus velezensis* V4, also was able to regulate the growth and development of multiple furuncles in aquaculture creatures [41]. Moreover, marine *Lactobacillus plantarum* AH 78 improved the immunity and performance of Nile tilapia through regulation of hepatic cytokine gene expressions, including interleukin-4, interleukin-12, and interferon-*γ* [42]. Likewise, our results demonstrated, for the first time, that marine probiotics, including *Enterococcus faecium* EA9, inhibit sepsis-induced pulmonary dysregulation and inflammation in CLP rats. In addition, the mechanistic pathways responsible for the reported protective effects were evaluated. *Enterococcus faecium* EA9 reduced oxidative stress and activated the antioxidant enzymatic activities. This could be through free radical scavenging possibilities. Lipid peroxidation was also suppressed in septic rats with *Enterococcus faecium* EA9 pretreatment suggesting reduced apoptosis and cellular damage as documented in the histological analysis. The ability of *Enterococcus faecium* EA9 to decrease the pulmonary inflammatory cytokines in CLP animals resulted in a marked reduction of pulmonary edema and enhanced architecture.

In conclusion, the marine isolate *Enterococcus faecium* EA9 showed antioxidant and anti-inflammatory properties and reduced lipid peroxidation in lung tissues of septic rats who underwent CLP surgeries. These beneficial properties were correlated with the restoration of pulmonary edema and lung injury. This study demonstrated clear evidence that probiotics developed from marine bioresources could represent a beneficial approach for inflammatory conditions, including sepsis.

## Figures and Tables

**Table 1:** Correlation analysis of inflammatory and oxidative stress biomarkers with lung injury score.

	TNF-*α*	IL-6	IL-1*β*	TBARS	GSH	CAT	SOD	GPx	GR
Pearson *r*	0.3612	0.5570	0.4377	0.4967	-0.3450	-0.6660	-0.4343	-0.3497	-0.2313
*p* value (two-tailed)	0.0829	0.0047	0.0324	0.0135	0.0987	0.0004	0.0339	0.0939	0.2768
*R* squared	0.1305	0.3103	0.1916	0.2467	0.1191	0.4436	0.1887	0.1223	0.05350

**Table 2:** Correlation analysis of inflammatory and oxidative stress biomarkers with pulmonary edema.

	TNF-*α*	IL-6	IL-1*β*	TBARS	GSH	CAT	SOD	GPx	GR
Pearson *r*	0.4603	0.4588	0.4854	0.6362	-0.2630	-0.3987	-0.5134	-0.4469	-0.4509
*p* value (two-tailed)	0.0236	0.0241	0.0162	0.0008	0.2143	0.0536	0.0103	0.0286	0.0270
*R* squared	0.2119	0.2105	0.2356	0.4047	0.06917	0.1589	0.2636	0.1997	0.2033

## Data Availability

The datasets generated and/or analyzed in the present study are included in the manuscript.
